# Impact of Dietary Galacto-Oligosaccharide (GOS) on Chicken’s Gut Microbiota, Mucosal Gene Expression, and *Salmonella* Colonization

**DOI:** 10.3389/fvets.2017.00192

**Published:** 2017-11-13

**Authors:** Rebecca-Ayme Hughes, Riawana A. Ali, Mary A. Mendoza, Hosni M. Hassan, Matthew D. Koci

**Affiliations:** ^1^Prestage Department of Poultry Science, North Carolina State University, Raleigh, NC, United States; ^2^Department of Chemistry, North Carolina State University, Raleigh, NC, United States

**Keywords:** chickens, immune, *Salmonella*, prebiotic, galacto-oligosaccharides

## Abstract

Preventing *Salmonella* colonization in young birds is key to reducing contamination of poultry products for human consumption (eggs and meat). While several *Salmonella* vaccines have been developed that are capable of yielding high systemic antibodies, it is not clear how effective these approaches are at controlling or preventing *Salmonella* colonization of the intestinal tract. Effective alternative control strategies are needed to help supplement the bird’s ability to prevent *Salmonella* colonization, specifically by making the cecum less hospitable to *Salmonella*. In this study, we investigated the effect of the prebiotic galacto-oligosaccharide (GOS) on the cecal microbiome and ultimately the carriage of *Salmonella*. Day-old pullet chicks were fed control diets or diets supplemented with GOS (1% w/w) and then challenged with a cocktail of *Salmonella* Typhimurium and *Salmonella* Enteritidis. Changes in cecal tonsil gene expression, cecal microbiome, and levels of cecal and extraintestinal *Salmonella* were assessed at 1, 4, 7, 12, and 27 days post infection. While the *Salmonella* counts were generally lower in the GOS-treated birds, the differences were not significantly different at the end of the experiment. However, these data demonstrated that treatment with the prebiotic GOS can modify both cecal tonsil gene expression and the cecal microbiome, suggesting that this type of treatment may be useful as a tool for altering the carriage of *Salmonella* in poultry.

## Introduction

*Salmonella* is a leading cause of foodborne disease in humans with poultry acting as a major source of human infection (1). Controlling *Salmonella* within poultry meat and egg production is critical to increase the safety of these products for human consumption. *Salmonella* infection in poultry is asymptomatic (2), so determining how young birds respond to *Salmonella* is important. Understanding their response will allow for the development of control methods that aid in the removal of *Salmonella* from poultry. While a number of *Salmonella* vaccines have been developed (3, 4), alternative control methods specifically targeting *Salmonella* within the bird’s intestinal tract may provide an effective method for reducing intestinal colonization. Prebiotics offer a potential intestinal *Salmonella* control strategy which can be added to feed and/or water without the need to modify the current production chain.

The reported top *Salmonella* serovars associated with human cases in 2015 were Enteritidis (20%), Newport (12%), and Typhimurium (11%) (5), and salmonellosis from poultry products are one of the top five causes of foodborne disease in the United States (6). Over the past 20 years, a great deal of effort has been spent to try and reduce the role poultry plays in salmonellosis, with some success (7, 8). Most of these efforts have targeted specific serovars, and consequently resulted in increased prevalence of other serovars increase in prevalence (9). Furthermore, different serovars respond differently to changes in the host as well as the host compartments they are associated with (10, 11). This highlights the need to develop methods that can effectively control colonization of multiple serovars.

*Salmonella* infection in birds is thought to be age dependent. Birds that are infected with *Salmonella* early in life (day 1 of life) carry *Salmonella* for an extended period, and in higher numbers, compared with birds infected at day 8 of life (12). Birds challenged earlier in life appear to clear reinfection slower than birds challenged later in life (3–6 weeks) (13). Exposure to *Salmonella* in the first 4 days post hatch has also been shown to result in detectable changes in the cecum microbiome (14). Modulation of the microbiome through the use of single administration of a *Salmonella* vaccine and or live probiotic (PrimaLac^®^) at day 1 of life has also been shown to have an effect on the gut microbiome development apparent from day 7 of life (15). However, *Salmonella* exposure at 16 days of life induced fewer changes in cecum microbiota (16). The modification of the microbiome occurring during early infection may allow the establishment of *Salmonella*.

The specialty feed additive market is projected to be worth over US$11 billion globally by 2022 (17). Prebiotics and probiotics represent a major component of this market and provide an alternative strategy, to the use of antibiotics as growth promoters (18, 19). The prebiotic galacto-oligosaccharides (GOS) has been shown to improve the intestinal architecture in the neonatal pig model along with improving the development of the microbiota and stimulating the intestinal defense mechanism (20), indicating that this prebiotic has significant beneficial properties. When fed to chickens, GOS in combination with the enzyme β-galactosidase has been reported to lead to an increase in *Bifidobacteria* and *Lactobacillus* (21). The presence of GOS within the intestinal tract has been shown to reduce the adherence and invasion of *Salmonella* in human enterocytes (22).

Treating birds with prebiotics offers a possible method for reducing *Salmonella* colonization through the modification of both the hosts’ immune system and the gut microbiome. The use of oligosaccharides extracts from palm kernels has been shown to improve the health status of broilers and reduce the levels of heterophils and basophils in circulation (23). Indeed, the addition of inulin as a prebiotic or *Lactobacillus lactis* subsp *lactis* 2955 *in ovo* has been shown to result in a reduction in the expression of IL-4, IL-6, IL-8, IL-12p40, and IL-18 in the cecal tonsil, with a reduction seen in the first 35 days after hatch (24). In other studies, *in ovo* administration of prebiotics and probiotics lead to upregulation of cytokine expression in the spleen and decreased expression in the cecal tonsil in birds at 6 weeks of age (25). To understand the effectiveness of prebiotic-supplemented diets on *Salmonella* control, it is important to determine the cecum immune response these treatments induce in poultry and the effect of a subsequent *Salmonella* challenge.

Preventing early infection of chicks is key to reducing the incidence of *Salmonella* in a flock. This study aimed to investigate the response of young birds to treatment with a prebiotic GOS on the carriage of *Salmonella*, cecal tonsil relative gene expression levels of markers of the immune response, and the cecal microbiome. This provides initial information on the effect of GOS on *Salmonella* control and modulation of the bird microbiome and immune response.

## Materials and Methods

### Ethics Statement

This study was carried out in strict accordance with the recommendations in the Guide for Care and Use of Laboratory Animals of the National Institutes of Health. All animals were maintained and euthanized according to a protocol no. 15-065-A approved by the Institutional Animal Care and Use Committee (OLAW no. D16-00214). All work was done in an approved biological safety level-2 laboratory or animal facility and appropriate personal protective equipment was used when handling *Salmonella*, a risk group two agent, or *Salmonella* infected birds.

### Birds and Experimental Details

Two hundred 1-day-old female Leghorn chickens (Hy-Line; Mansfield, GA, USA) were split into two treatment groups (control and prebiotic). Birds were provided with *ad libitum* food and water with the prebiotic group receiving feed supplemented with 1% functional GOS [1.8% w/w of commercial GOS (Oligomate™ 55NP) (Kanematsu; Somerset, NJ, USA) that contained 55–56% GOS and 44–45% monosaccharides]. The control feed was supplemented with 0.8% glucose to control for the monosaccharides present in the commercial GOS. Birds were housed in 934-1-WP isolators (Federal Designs Inc.; Comer, GA, USA) with regulated temperatures, airflow, 12/12 light/dark cycle, wire flooring, with free access to feed and water. Half of the birds from each treatment group were challenged with100 μL containing a mixture of 5.5 × 10^8^ CFU of rifampicin-resistant (Rif^R^) isolate of *S*. Typhimurium FNR-HA—kanamycin-resistant (Kan^R^) ATCC 14028s (26) and 6.6 × 10^8^ CFU *S*. Enteritidis ATCC 31194 modified to express FNR-HA—chloramphenicol-resistant (Cm^R^) and Rif^R^ (unpublished) in 100 µL of phosphate-buffered saline (PBS) per bird by oral gavage at day 3 of life; non-challenged birds were given 100 µL of PBS. Challenged birds were housed separately from their non-challenged counterparts. At 1, 4, 7, 12, and 27 days post infection (dpi), eight birds per treatment group were randomly selected, euthanized, and samples of cecum contents, and liver were collected for microbiological analysis, and cecal tonsil collected to assess the effect of diet and *Salmonella* infection on host gene expression.

### *Salmonella* Preparation for Gavage

Overnight cultures of *S*. Typhimurium ATCC 14028s—Kan^R^ Rif^R^ (ST) and *S*. Enteritidis ATCC 31194—Cm^R^ Rif^R^ (SE) were prepared individually from glycerol stocks in LB (Luria-Bertani) broth (Thermo Fisher Scientific; Waltham, MA, USA) incubated at 37°C without shaking. Antibiotics were added at the following concentrations: Kan, 50 µg/mL; Cm, 20 µg/mL; and Rif, 100 µg/mL. Cells were centrifuged at 8,000× *g* for 15 min and washed three times in PBS with 2-mM magnesium sulfate. Optical density (OD) was determined at 600 nm using a BioRad Smartspec 3000 (BioRad; Hercules, CA, USA) with a 1-cm light path, and adjusted to an OD600 of 10 for ST and 20 for SE (equivalent to ~1,010 CFU/mL of each serovar, according to a standard predetermined relationship between OD600 and viable cell counts). ST and SE cultures were mixed 1:1 just prior to inoculating the chicks. The actual concentration of the bacteria in the gavage mixture was determined by plating a serial dilution on XLT4-Agar (Xylose-Lysine-Tergitol4—Neogen; Lansing, MI, USA) containing the appropriate antibiotics. Challenged birds were given 100 µL of *Salmonella* solution by oral gavage using gavage needles (Thermo Fisher Scientific); control birds were given 100 µL of PBS by oral gavage.

### Sample Collection and Preparation

Cecal tonsil tissue, a visible nodule of lymphoid tissue at the proximal end of chicken ceca, was removed and immediately snap frozen in liquid nitrogen and stored at −80°C until processed. Cecum contents were collected from each bird for microbiome and bacteriological analyses. Samples collected for bacteriological analysis were weighed individually and resuspended in 500 µL PBS + 25% glycerol 2-mM MgSO_4_, and stored at −80°C. Content was serially diluted and plated on XLT4 + 100-mM MOPS (pH 7.4) with relevant antibiotics (ST = Kan, SE = Cm) to determine *Salmonella* levels. The CFU/g of cecum content was determined based on the weight of sample and the volume of PBS. Up to 0.5 g of liver were removed and individually placed in 1 ml PBS + 25% glycerol 2-mM MgSO_4_ Liver samples were weighed, resuspended to 100 mg/ml and homogenized using a Bio-Gen Pro-200 homogenizer (Pro Scientific Inc.; Oxford, CT, USA). For liver samples that were *Salmonella* negative on XLT4 plates, a volume of a homogenized sample equivalent to 500 mg of liver was enriched in Rappaport—Vassiliadis media (Difco) for 24 h and streaked on XLT4 + 100-mM MOPS (pH 7.4) supplemented with the relevant antibiotics for the detection of SE and ST as stated above. *Salmonella* colonies were confirmed by their resistance to the appropriate antibiotic markers, formation of black colonies on XLY4 plates, and biochemical tests using API- 20E system (Biomerieux; Durham, NC, USA).

### RNA Extraction

RNA was extracted from 30 to 50 mg of snap frozen cecal tonsil tissue from each bird in each treatment group and time point. Tissue was homogenized using 2.8-mm ceramic beads (Qiagen; Valencia, CA, USA) and RNA extracted using Nucleospin^®^ RNA kit (Macherey-Nagel; Bethlehem, PA, USA) following the kit protocol with the exception that the RNA was eluted in 50 µL of H_2_O. RNA was quantified using a NanoDrop 2000 spectrophotometer (NanoDrop, Wilmington, DE, USA) and then stored at −80°C until needed.

### cDNA and Real-Time Polymerase Chain Reaction for Individual Birds

cDNA was made using the High-Capacity cDNA Reverse Transcriptase Kit (Thermo Fisher Scientific) from 1 µg of extracted RNA from each of five birds from each treatment group and time point. Real-time polymerase chain reaction (RT-PCR) was carried out on in triplicate wells for each sample using the ABI Taqman PCR assays listed in Table 1. All FAM-labeled gene expression assays for target genes, TaqMan Universal Master mix II, and VIC-labeled Euk 18S rRNA endogenous housekeeping control assay were purchased from Thermo Fisher Scientific. For each sample and each gene of interest, the cDNA was diluted 1:40 in ultrapure water and added to the reaction mix containing 1 µL of gene of interest primer/probe and 1 µL of endogenous control primer/probe; all reactions were carried out in triplicate. RT-PCR was run on an ABI Step-One Plus (Thermo Fisher Scientific) using the standard program (Holding stage: 50°C for 2 min, 95°C for 10 min, Cycle stage: (40 cycles) 95°C for 15 s, 60°C for 1 min). Thresholds were set for both housekeeping and gene of interest set the same within each plate. Each gene was assayed on one plate per time point for all conditions. The ΔCT was calculated (CT of gene of interest—CT of housekeeping gene) for each triplicate well, and an average ΔCT calculated for each RNA sample. The non-treated-non-challenged control RNA sample with the median ΔCT from among the five replicate samples was used as the reference sample for each plate. The ΔΔCT was then determined for all samples [ΔΔCT = ΔCT (average ΔCT of untreated-non-challenged control) − ΔCT (each sample)], such that positive ΔΔCT denotes increased expression and negative ΔΔCT denotes decreased expression. Results are plotted as ΔΔCT which is equal to the log_2_ 2^ΔΔCT^. Statistical analysis of the resulting data was carried out using Prism 7.0c (GraphPad Software, Inc.; La Jolla, CA, USA). Differences in *Salmonella* log_10_ colony forming units, or the ΔΔCT between treatment groups was assessed using a two-way analysis of variance (ANOVA) followed by a Tukey’s multiple comparisons test.

**Table 1:** Assays used in real-time PCR.

Gene	ABI assay ID	Label
MAPK1	Gg03363520_m1	FAM
MAPK14	Gg03323838_m1	FAM
JUN	Gg03356263_s1	FAM
FASLG	Gg03353844_m1	FAM
TLR4	Gg03354643_m1	FAM
MYD88	Gg03355572_m1	FAM
IRF7	Gg03339761_g1	FAM
INFB	Gg03344129_s1	FAM
18S	431913E	VIC

*All MGB probes were obtained at 20× working concentration from Thermo Fisher Scientific*.

*PCR, polymerase chain reaction*.

### Analysis of Network Determination and Interaction

The pathway network of the eight genes focused on in this study and their connecting genes was determined using Pathway Commons^1^ and using neighborhood as the interaction.

### Microbiome Analysis

Cecum contents were collected from each bird into duplicate tubes, snap frozen in liquid nitrogen, and stored at −80°C until processed. Isolation of total genomic DNA and microbiome sequencing was performed by Microbiome Core Facility at UNC^2^. Briefly DNA was isolated using a Qiagen BioRobot Universal instrument (Qiagen) and the E.Z.N.A. Stool DNA Kit (Omega Bio-Tek, Norcross, GA, USA) according to the manufacturer’s specifications. DNA was quantified using a NanoDrop 2000 spectrophotometer (NanoDrop). Amplicons generated from regions V1–V2 of the 16S rRNA gene were sequenced on the Ion Torrent PGM sequencing platform from Life Sciences.

Processing and analysis of sequencing data was done using the Qiagen CLC Genomics Workbench Version 10.1 and the Microbial Genomics Module. Briefly, raw Fastq files were demultiplexed and reads were trimmed to 98 bp. Operational taxonomic units (OTUs) were clustered using the reference Greengenes v13_5 99% database. Chimeras and OTUs with low abundance (less than 10 reads) were removed. Bray–Curtis dissimilarity analysis and permutational multivariate analysis of variance (PERMANOVA) were performed. Statistical analysis of the changes in microbiome was carried out using Prism 7.0c (GraphPad Software, Inc.). Differences in microbiome between control- and prebiotic-treated groups at each time point were assessed using two-way ANOVA using Sidak’s multiple comparison test to determine significant difference at the family level. Data presented are the average of eight animals per treatment and time point.

## Results

### *Salmonella* Typhimurium More Persistent at Invading and Colonizing the Chicken than *Salmonella* Enteritidis

At 1, 4, 7, 12, and 27 dpi, animals were sacrificed and samples collected from the liver and cecum to assess the level of *Salmonella* invasion of systemic tissues, and colonization of the cecum. The results demonstrated that only ST invaded the liver, but only in a few animals, and only between days 4 and 12 post infection (Table 2). There was no significant difference (α = 0.05) in the numbers of ST positive liver samples between the birds that received the control diet and those fed the prebiotic.

**Table 2:** *Salmonella* invasion of the liver—number of positive birds/total challenged.^a^

dpi	Control		Prebiotic	
	*Salmonella* Typhimurium	*Salmonella* Enteritidis	*Salmonella* Typhimurium	*Salmonella* Enteritidis
1	0/8	0/8	0/8	0/8
4	2/8	0/8	1/8	0/8
7	3/8	0/8	2/8	0/8
12	1/8	0/8	1/8	0/8
27	0/8	0/8	0/8	0/8

*^a^At the specified time points (dpi), eight birds from each treatment were euthanized; liver samples were collected, homogenized, and tested for the presence of ST and SE as described in Section “Materials and Methods.”*

**Figure 1:**
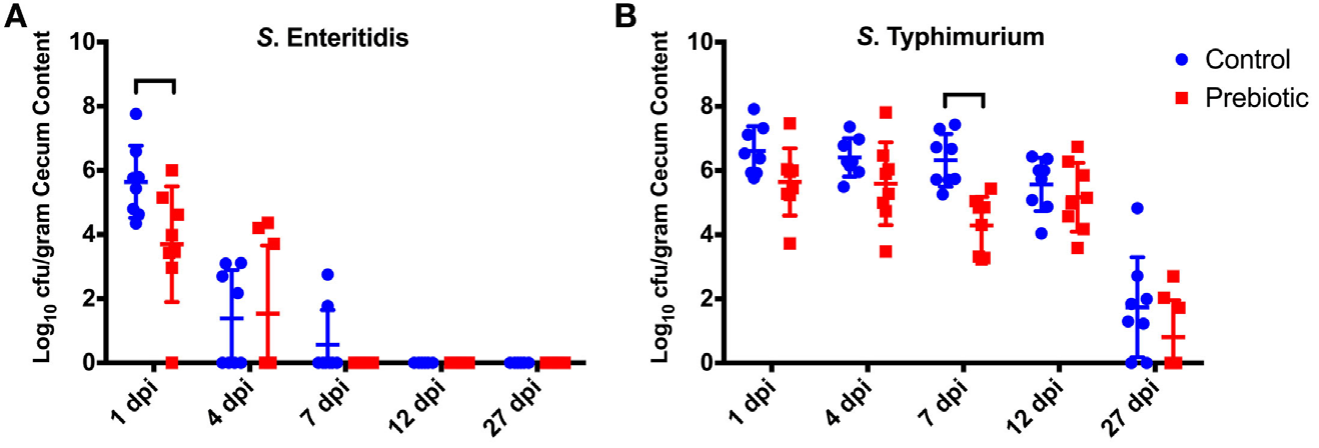
Effect of prebiotic diet on *Salmonella* colonization of the cecum. Birds were fed a conventional diet (Control) or fed a diet containing 1% GOS (Prebiotic). Birds were then infected with 6.6 × 10^8^ CFU SE and 5.5 × 10^8^ CFU of ST by oral gavage at 3 days of age. The levels of SE **(A)** and ST **(B)** were assessed in the cecum at 1–27 days post infection (dpi). The CFU/g of cecal contents is shown for each individual bird sampled at each time point, mean represented by lines. Brackets denote time points where the means were significantly different (*p* < 0.05). GOS, galacto-oligosaccharide.

The observation that ST was better able to survive within the chicken’s liver was also observed in the cecum (Figure 1). While SE and ST were detected in the cecum of both control and prebiotic, there was a difference in the number of positive birds between treatment groups; this was more apparent within the SE infection levels. SE was detectable in 7/8 prebiotic birds at 1 dpi, 3/8 at 4 dpi, and was undetectable by 7 dpi, whereas in control birds SE was detected in 8/8 birds at 1 dpi, 4/8 at 4 dpi, 2/8 at 7 dpi, and then undetectable by 12 dpi (Figure 1A). However, ST was able to persist in the cecum much longer (Figure 1B). Birds from both the control and prebiotic treatment groups remained positive at 27 dpi with 6/8 positive birds in the control group and compared with 3/8 in the prebiotic group. In the case of SE the prebiotic-treated birds had significantly lower CFU/g cecum content at 1 dpi compared with the control birds. The prebiotic birds also had significantly lower ST CFU/g cecum content at 7 dpi. No other time points showed significant differences in the carriage of *Salmonella* between treatment groups (Figure 1).

### Host Response to *Salmonella* Infection

**Figure 2:**
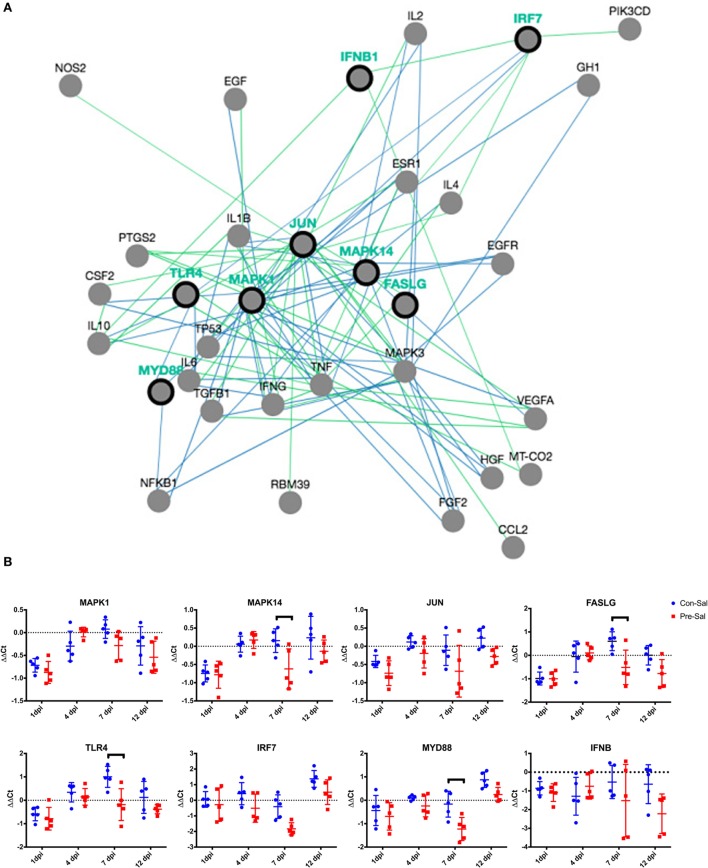
Difference in relative gene expression following *Salmonella* infection between birds fed control or prebiotic diets. **(A)** Gene pathway network showing the eight genes assayed (MAPK1, MAPK14, JUN, FASLG, TLR4, IRF7, MYD88, IFNB, represented by black ringed dots) and 18 nearest neighbors (represented by gray dots). Blue lines denote regulation of protein state. Green lines denote regulation of expression. Network was produced using PCViz (http://www.pathwaycommons.org/). **(B)** Birds were either fed a standard diet (Con) or fed a diet containing GOS (Pre). Birds were infected with SE and ST by oral gavage at 3 days of age (Sal). The ΔΔCt for each individual bird sampled at each time point is represented by blue circles (Con–Sal) or red squares (Pre–Sal). The sample mean is represented by the horizontal line, with upper and lower error bars denoting 1 SD among the five replicate samples. The dashed line denotes no change relative to the control reference sample. Data points above the dashed line represent increased expression, while points below denote decreased expression. Black brackets denote time points where the means were significantly different (*p* < 0.05). GOS, galacto-oligosaccharide.

RNA was collected from the cecal tonsils from chicks at 1, 4, 7, and 12 dpi and analyzed for changes in gene expression of MAPK1, MAPK14, JUN, FASLG, TLR4, IRF7, MYD88, and IFNB (Figure 2A). Initially samples were compared with identify differences in gene expression between prebiotic- and control-fed animals following *Salmonella* infection. This comparison demonstrated significant (*p* < 0.05) decreases in expression of MAPK14, FASLG, TLR4, and MYD88 at 7 dpi in the prebiotic-treated group (Figure 2B). A decrease in the expression was also seen in JUN (*p* = 0.051) and interferon regulatory factor 7 (IRF7) (*p* = 0.067) at 7 dpi in the prebiotic-treated birds.

**Figure 3:**
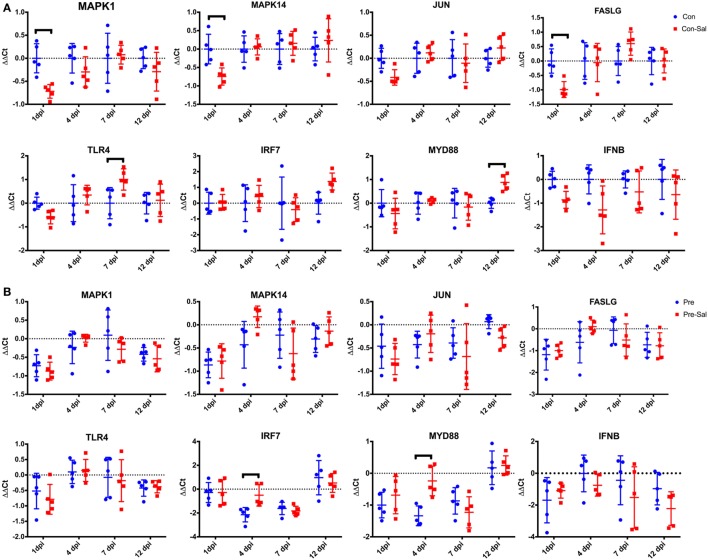
Changes in cecal tonsil gene expression in birds following infection with SE and ST. Birds were fed a conventional diet (**A**, Con) or a prebiotic diet containing GOS (**B**, Pre). At 3 days of age, birds were infected with SE and ST (Sal) or mock infected with PBS. At 1, 4, 7, and 12 dpi, the cecal tonsils were collected from five birds bird treatment group and assayed for relative changes in gene expression. The ΔΔCt for each individual bird sampled at each time point is represented by blue circles (mock infected) or red squares (*Salmonella* infected). The sample mean is represented by the horizontal line, with upper and lower error bars denoting 1 SD among the five replicate samples. The dashed line denotes no change relative to the control reference sample. Data points above the dashed line represent increased expression while points below denote decreased expression. Black brackets denote time points where the means were significantly different (*p* < 0.05). GOS, galacto-oligosaccharide; PBS, phosphate-buffered saline.

To understand the effect of GOS treatment on *Salmonella* challenge, the fold change in gene expression between the uninfected and infected animals within in each diet group was determined. This analysis demonstrated that in the control-fed animals, *Salmonella* infection led to a transient downregulation of MAPK1, MAPK14, and FASLG at 1 dpi (*p* < 0.05, Figure 3A). This is followed by an increase in expression (*p* < 0.05) of TLR4 at 7 dpi and MYD88 at 12 dpi in the control non-challenged group (Figure 3A). The expression of IRF7 increases at 12 dpi but this is not significant (*p* = 0.083). Comparatively, there are fewer changes in gene expression between the uninfected and infected animals fed the prebiotic diet (Figure 3B). There was a significant (*p* < 0.05) increase in the expression of IRF7 and MYD88 in the infected animals at 4 dpi. The expression levels of MAPK14 also increase at 7 dpi; however, this is not significant (*p* = 0.073) (Figure 3B).

**Figure 4:**
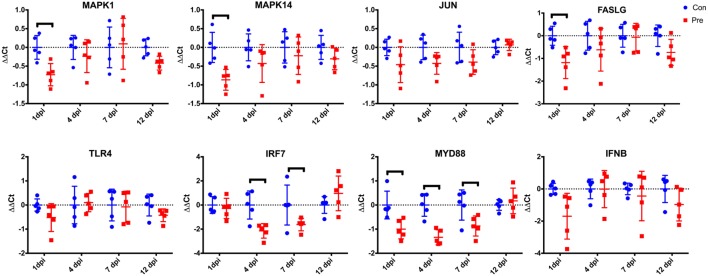
Effect of prebiotic diet on cecal tonsil gene expression in uninfected birds. Birds were fed a conventional diet (Con) or a prebiotic diet containing GOS (Pre). At 3 days of age, birds were mock infected with PBS and the cecal tonsils were collected from five birds bird per treatment group at 1, 4, 7, and 12 dpi. Samples were assayed for relative changes in gene expression. The ΔΔCt for each individual bird sampled at each time point is represented by blue circles (Con) or red squares (Pre). The sample mean is represented by the horizontal line, with upper and lower error bars denoting 1 SD among the five replicate samples. The dashed line denotes no change relative to the control reference sample. Data points above the dashed line represent increased expression, while points below denote decreased expression. Black brackets denote time points where the means were significantly different (*p* < 0.05). GOS, galacto-oligosaccharide; PBS, phosphate-buffered saline.

To further assess the effect of the prebiotic on the host immune response, the difference in expression of these genes between the uninfected control-fed and prebiotic-fed animals was assessed (Figure 4). This comparison shows a significant (*p* < 0.05) decrease in expression of MAPK1, MAPK14, FASLG, and MYD88 at 1 dpi. At 4 dpi IRF7 and at 4 and 7 dpi, MYD88 showed a significant decrease in expression (note these were not infected, but age-matched birds Figure 4).

### GOS and *Salmonella* Affecting Microbiome Structure

**Figure 5:**
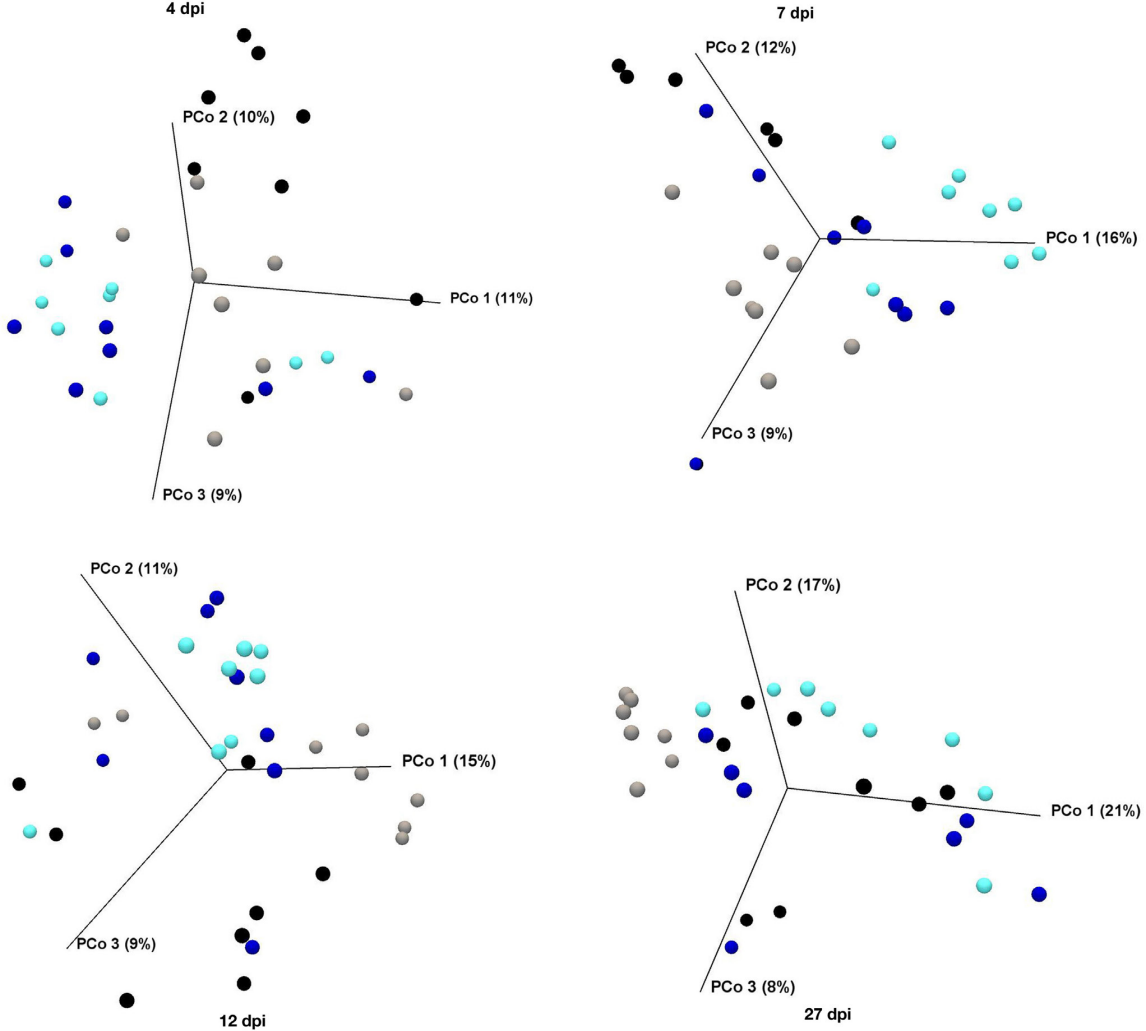
PCoA using OTU-level Bray–Curtis index of the cecal microbiome showing beta diversity following *Salmonella* infection between birds fed control or prebiotic diets. Birds were either untreated (Con, gray dots) or fed a diet containing GOS (Pre, teal dots). At 3 days of age, half of the birds in each diet group were infected with SE and ST by oral gavage (Con-Sal, black dots or Pre-Sal, blue dots). At 4, 7, 12, and 27 dpi, cecal contents were collected for microbiome analysis. GOS, galacto-oligosaccharide; PCoA, principal component analysis; OUT, operational taxonomic units.

The analysis of the diversity of the cecal microbiome demonstrated that the prebiotic treatment results in a significantly (*p* < 0.004) diverse population as compared with the control-fed animals (Figure 5; Table 3). The microbiomes of the uninfected control and uninfected prebiotic groups were found to be significantly diverse (*p* < 0.004) at each time point examined. Interestingly, the uninfected control group is the only group whose microbiome is significantly distinct (*p* < 0.02) from all other groups for the duration of the experiment. Conversely, the microbiomes of the uninfected prebiotic group and the infected prebiotic group were found to be significantly diverged (*p* < 0.02) at 4, 7 and 12 dpi, but by 27 dpi there was no significant difference (Figure 5; Table 4), in spite of the fact that ST was found in the cecum of the infected birds in both control and GOS diet groups (Figure 1). Interestingly, the diversity of the microbiome of infected control and infected prebiotic birds was only significant at 4 dpi (*p* < 0.003).

**Table 3:** PERMANOVA analysis (Bray–Curtis) showing statistical differences in the beta diversity measures between the different treatments.^a^

		*p*-Value (Bonferroni)			
		4 dpi	7 dpi	12 dpi	27 dpi
Control	Control–Challenge	0.00653	0.00559	0.00280	0.00839
Control	Prebiotic	0.00373	0.00093	0.00093	0.00093
Control–Challenge	Prebiotic	0.00466	0.00186	0.00932	0.09604
Control	Prebiotic–Challenge	0.00373	0.00746	0.01399	0.00373
Control–Challenge	Prebiotic–Challenge	0.00280	0.35897	0.05594	0.62378
Prebiotic	Prebiotic–Challenge	0.00373	0.00093	0.01678	0.55664

*^a^p ≤ 0.05 are significant and p ≤ 0.005 are highly significant*.

**Table 4:** Taxonomic analysis of the cecum micobiome composition of prebiotic and control-treated non-challenged birds.

Taxonomy	Day 4		Day 7		Day 12		Day 27	
	Control	Prebiotic	Control	Prebiotic	Control	Prebiotic	Control	Prebiotic
Bacteria	0.0000	0.0000	0.0015	0.0000	0.0052	0.0001	0.0014	0.0004
Cyanobacteria, Chloroplast, Streptophyta	0.0027	0.0046	0.0012	0.0001	0.0000	0.0004	0.0037	0.0000
Firmicutes	0.0014	0.0016	0.0081	0.0007	0.0026	0.0004	0.0035	0.0001
Firmicutes, Bacilli, Bacillales, Bacillaceae	0.0078	0.0000	0.0088	0.0001	0.0188	0.0290	0.0260	0.0360
Firmicutes, Bacilli, Lactobacillales, Lactobacillaceae	0.1260	0.0975	0.1026	0.0250****a	0.0086	0.2403****b	0.0070	0.2708****b
Firmicutes, Clostridia, Clostridiales	0.1002	0.0888	0.1201	0.0958	0.0746	0.0791	0.0728	0.0509
Firmicutes, Clostridia, Clostridiales	0.1673	0.0602****a	0.1493	0.0978	0.1128	0.1985****b	0.1599	0.0886
Firmicutes, Clostridia, Clostridiales, Clostridiaceae	0.0009	0.0105	0.0005	0.0056	0.0011	0.0045	0.0030	0.0032
Firmicutes, Clostridia, Clostridiales, Lachnospiraceae	0.2798	0.4958****b	0.2631	0.3974****b	0.1488	0.1930*b	0.2170	0.1315****a
Firmicutes, Clostridia, Clostridiales, Peptostreptococcaceae	0.0008	0.0048	0.0000	0.0020	0.0000	0.0004	0.0043	0.0001
Firmicutes, Clostridia, Clostridiales, Ruminococcaceae	0.1764	0.1394***a	0.2749	0.3221	0.5381	0.1962****a	0.3849	0.2785***b
Firmicutes, Erysipelotrichi, Erysipelotrichales, Erysipelotrichaceae	0.0501	0.0148	0.0082	0.0207	0.0303	0.0171	0.0556	0.0500
Proteobacteria, Gammaproteobacteria	0.0016	0.0082	0.0001	0.0007	0.0002	0.0059	0.0002	0.0000
Proteobacteria, Gammaproteobacteria, Enterobacteriales, Enterobacteriaceae	0.0187	0.0570	0.0007	0.0087	0.0008	0.0174	0.0018	0.0004
Tenericutes, Mollicutes, Anaeroplasmatales, Anaeroplasmataceae	0.0000	0.0000	0.0002	0.0000	0.0063	0.0000	0.0012	0.0005
Tenericutes, Mollicutes, RF39	0.0001	0.0000	0.0041	0.0000	0.0078	0.0000	0.0033	0.0000
N/A	0.0609	0.0095	0.0542	0.0229	0.0414	0.0167	0.0510	0.0873

Statistical differences between control and prebiotic non-challenged age-matched birds using two-way analysis of variance (ANOVA) using Sidak’s multiple comparison test. ****p < 0.0001, ***p < 0.006, **p < 0.01, and *p < 0.05. A decrease in abundance in the prebiotic group is indicated by “a” and an increase is indicated by “b.”

**Figure 6:**
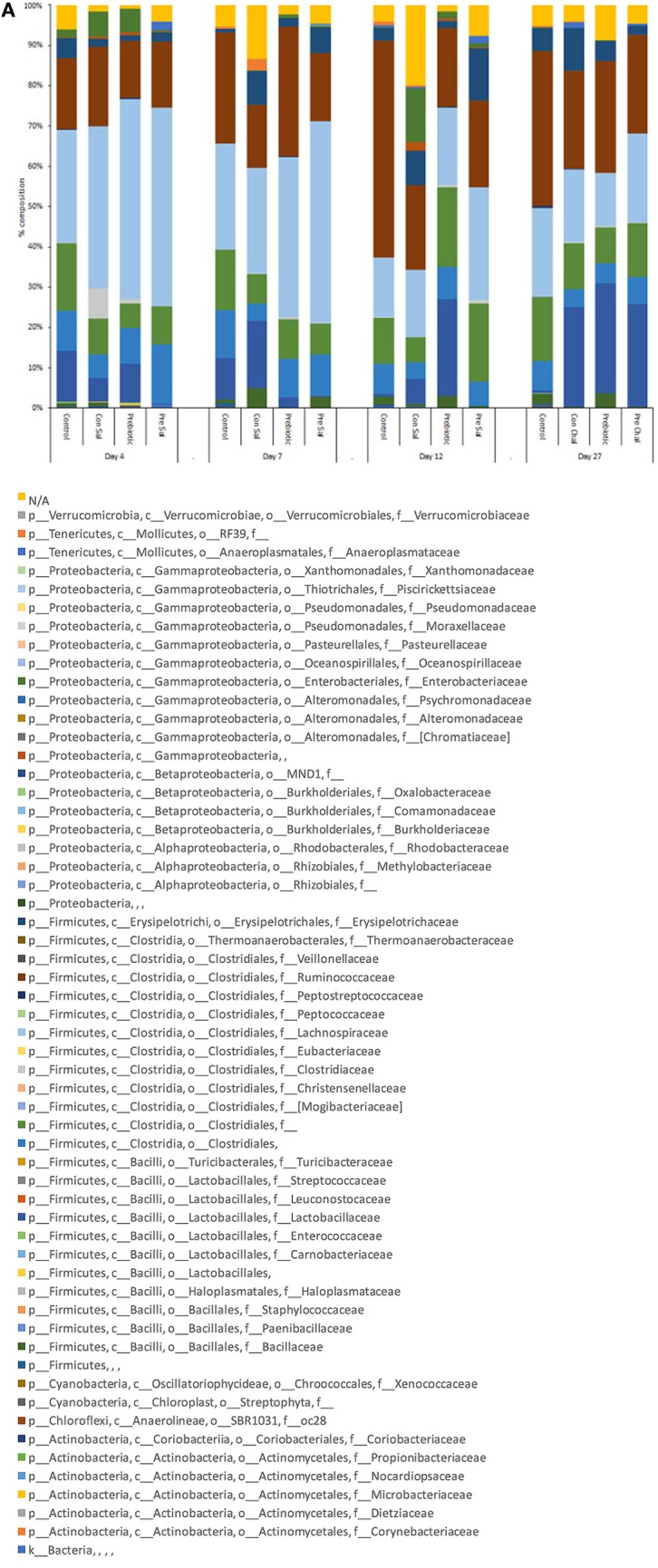

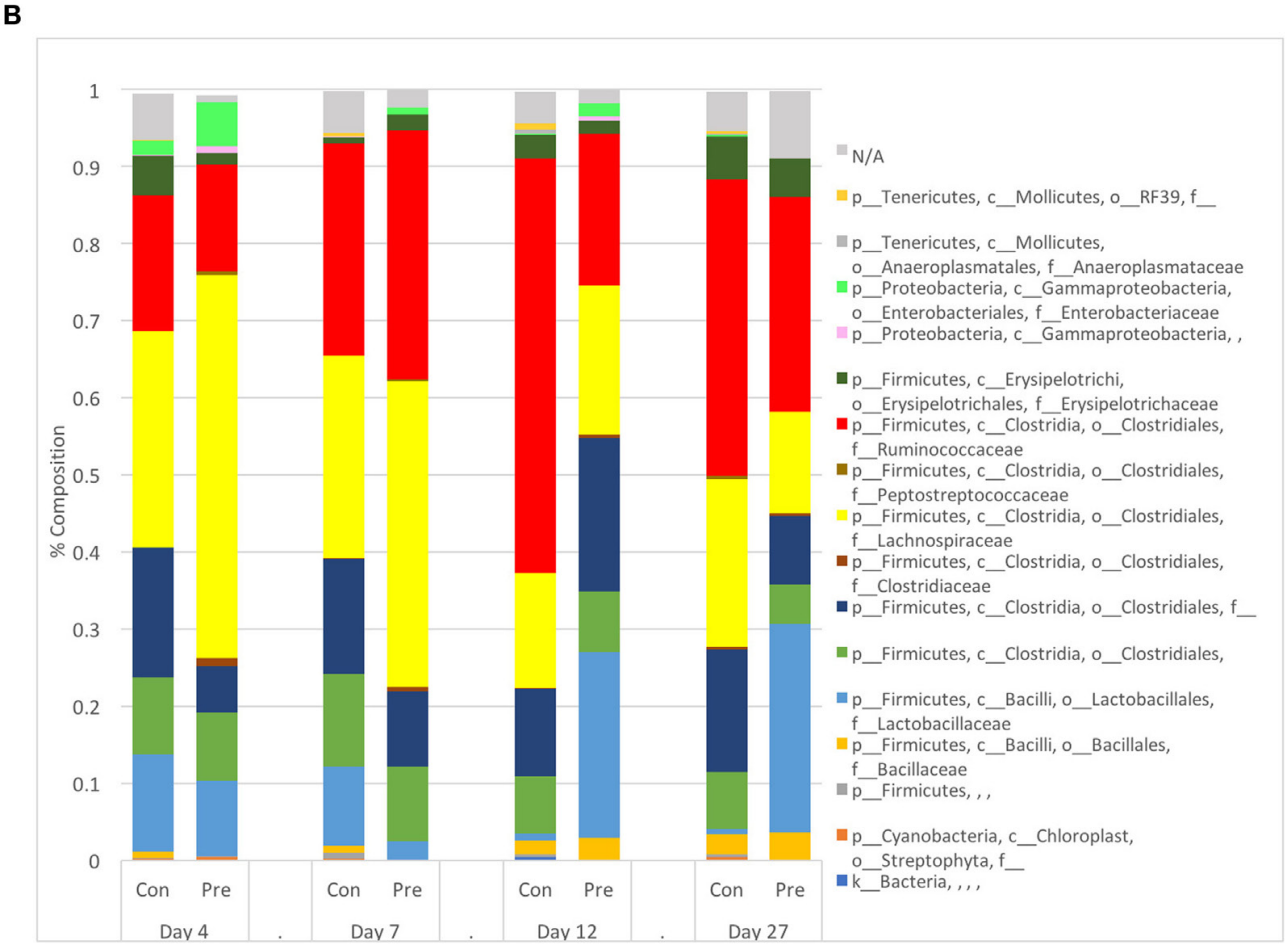
Average relative abundance of the microbiota at the family level in ceca as **(A)** function of time following *Salmonella* infection in birds fed control or prebiotic diets or **(B)** function of time and diet composition. Birds were either untreated (Con) or fed a diet containing GOS (Pre). At 3 days of age, half of the birds in each diet group were infected with SE and ST by oral gavage (Sal). At 4, 7, 12, and 27 dpi, cecal contents were collected for microbiome analysis as stated in Section “Materials and Methods.” GOS, galacto-oligosaccharide.

Analysis of the effect of diet treatment and *Salmonella* challenge on the family level taxonomic changes of the microbiota in each of the treatment groups demonstrated that by 27 dpi there is between 30- and 50-fold more *Lactobacillaceae* in the prebiotic, prebiotic-challenge, and control-challenged groups as compared with the control group (Figure 6A). Conversely, the control group contains more members of the order *Clostridiales* than the other three groups (Figure 6A).

A further in-depth analysis focusing on the differences in microbiome composition between the prebiotic and control-treated and non-infected age-matched animals at each time point indicated statistically significant differences between the two treatments at all time points investigated. In age-matched GOS-treated birds, there was a general decrease in *Clostridiales, Lachnospiraceae*, and *Ruminococcaceae* and increase in *Lactobacillaceae* over time as compared with the control birds over time. Specifically, at 4 dpi there was a significant decrease in *Clostridia Clostridiales*, and *Clostridiales Ruminococcaceae* and an increase in *Clostridiales Lachnospiraceae* in the GOS-treated birds compared with the control-treated birds (Table 4; Figure 6B). At 7 dpi, the levels of *Lactobacillales Lactobacillaceae* were significantly decreased and *Clostridiales Lachnospiraceae* was significantly increased in the GOS-treated birds compared with the control-non-treated birds (Table 4; Figure 6B). At 12 dpi, there was a significant decrease in the levels of *Clostridiales Ruminococcaceae* and a significant increase in the levels of *Lactobacillales Lactobacillaceae, Clostridia Clostridiales*, and *Clostridiales Lachnospiraceae* in GOS-treated birds compared with control-treated birds (Table 4; Figure 6B). At 27 dpi, the levels of *Clostridiales Lachnospiraceae* and *Clostridiales Ruminococcaceae* were significantly decreased and the levels of *Lactobacillales Lactobacillaceae* were significantly increased in GOS-treated birds as compared with control-treated birds (Table 4; Figure 6B).

## Discussion

Data from this study indicated that there is a difference in the colonization capabilities of the ST and SE strains used. ST demonstrated the greatest degree of persistence within the cecum of control birds; at 27 dpi it was still detectable in six out of eight control birds, whereas SE was undetectable by 12 dpi. The difference in persistence and cecum colonization between ST and SE seen in this study has also been noted by other researchers using a coinfection with SE and ST in 1-day-old chicks (26). Similar differences in competitive fitness have also been reported between *S*. *Kentucky* and ST in coinfected chickens *S. Kentucky* and ST/SE (27, 28). Furthermore, challenging birds prior to molt with a non-SE serovar before exposure to SE has been shown to reduce SE-associated problems during molt (29), indicating that delayed coinfection in older birds also results in out competition of SE. The reasons for the differences in persistence between the serovars is unclear; it is possible that the intestinal environmental conditions (microbiota, immune response) within the bird plays a significant role in determining which serovar is able to establish within the cecum and remain detectable for 27 dpi. The addition of GOS to the chicken feed resulted in a minimal change in the rate of clearance of ST and SE, with SE being undetectable at day 7 in prebiotic treated but day 12 in control birds. An increase in the rate of clearance of *Salmonella* has also been reported in birds treated with the prebiotics mannan-oligosaccharides (MOS) or xylooligosaccharides (XOS) (30). Clearly, understanding the competitive fitness of different serovars, and the role prebiotics such as GOS play in modulating the cecal environment (microbiome, pH, and host factors), will enable a better determination of conditions needed to reduce *Salmonella* colonization in young birds.

Vaccination of birds against *Salmonella* has been the dominant control strategy. Commercial vaccines have been shown to provide protection against a *Salmonella* challenge by inducing an IgG response (4). While there is evidence that vaccines can induce a serum IgG response and an intestinal IgA response, the level of this response is dependent on the vaccine schedule with a combination of live and killed vaccines leading to a higher level of IgA response (31). Such a response would be required to protect the intestine from *Salmonella* colonization. However, the IgG response has been shown to be serovar specific with an SE vaccine failing to provide protection against an ST or *S*. *Heidelberg* challenge (32). It is reasonable to hypothesize that the IgA response induced by the vaccine would also be serovar specific. The use of serovar specific vaccines has also been shown to allow a switch in the dominant serovar present in the chickens; thus, the eradication of *S*. *gallinarum* through the use of vaccines leads to an increase in colonization by SE (33). The other switches in serovar dominance have also been seen in chickens and in pigs (34). Modulating the chicken intestinal microbiology through *Salmonella* serovar specific exclusion appears to be a method of modulating the serovar of *Salmonella* carriage, and further methods of modulation accompanied by the use of vaccines may allow more robust removal of *Salmonella* from the chicken.

Taking into account the transient presence of ST in the liver (Table 2) liver samples from *Salmonella* infected chicks demonstrated that ST was able to transiently infect the liver, indicating that it was able to cross the intestinal barrier whereas SE was not able to do so. The infection of ST in the liver was short lived and only detectable between 4 and 12 dpi (Table 2) and long with the drop in SE cecum levels seen between 1 and 7 dpi (Figure 1A). Coupled with the lack of detectable IgG response (data not shown) suggests a role for both the chicks innate immune system and microbiota in reducing the systemic infection.

The prebiotic MOS has been reported to reduce the expression of TNFα and IFNγ in the cecal tonsil of young birds challenged with *Salmonella* (30); and Fructo-oligosaccharides (FOS) inulin has been shown to reduce the expression of IL-1β in the chicken macrophage HD11 cell line when *Salmonella* challenged (35). The genes analyzed in this study were selected from RT-PCR gene expression array panels containing genes related to the antibacterial immune response and inflammation. The eight target genes (MAPK1, MAPK14, JUN, FASLG, TLR4, IFR7, MYD88, and IFNB) represent genes from a cross-section of receptors, ligands, and intracellular signaling factors related the antibacterial response. These genes were selected due to their differential expression in cecum tissues in the presence and absence of *Salmonella* (data not shown) and therefore allow the detection of bird immune response under the conditions reported in this study. Challenged birds treated with prebiotic (GOS) had a significantly lower expression (4/8 genes at 7 dpi Figure 2B) than control challenged birds. This is consistent with other studies using prebiotics in both young birds and cell culture (30, 35). At 7 dpi, there was a decrease in the levels of MAPK14 and FASLG in the prebiotic-treated birds along with TLR4 and MYD88, suggesting that both these pathways are involved in the response. This marked difference in gene expression between the two treatment groups may indicate a switch in response pathway induced through changes in the microbiome or changes in the host immune cells repertoire presented in the cecum lumen and cecal tonsil.

The response within each treatment group between challenged and non-challenged birds (Figures 3A,B) indicates that there is a greater degree of gene changes within the control group, with a reduction in gene expression seen at 1 dpi with 3/8 genes and an increase seen in one different gene at both 7 and 12 dpi. The downregulation of genes at 1 dpi occurring in the MAPK–FASLG pathways prior to an increase in TLR4 (7 dpi) and MYD88 (12 dpi) indicates that initially the response to *Salmonella* in the control birds is modulated through a downregulation of the MAPK-FASLG pathway (Figure 2A). While the prebiotic challenge group underwent an increase in the expression of IRF7 and MYD88 at 4 dpi, this is a different mechanism of response compared with the control challenged birds. The mechanism behind this altered immune response is currently unclear, and further studies are needed to understand the signals that led to this change in response, along with determining the response of the other genes contained within these two pathways in response to *Salmonella* in the presence and absence of prebiotic.

Interestingly, age-matched non-challenged prebiotic birds had a significant reduction in 4/8 genes at 1 dpi, 2/8 at 4 dpi, and 1/8 at 7 dpi, indicating a downregulation of genes in the MYD88-IRF7 pathway occurring at 4 and the FASLG-MAPK occurring at the time point equivalent to 1 dpi. A similar response was seen in cecal tonsil cytokine expression when the prebiotic inulin was given *in ovo* to chickens (24). These data suggest that feeding GOS can modulate the bird’s immune response and in turn potentially change the cecum environment (microbiome, pH, host factors) within the bird.

Myeloid differentiation primary response protein 88 (MYD88) has been shown to interact with IRF7 leading to the induction of INF? and INF inducible genes in heterophils exposed to *Salmonella* LPS (36). The downregulation of the gene encoding for these proteins in the prebiotic and prebiotic challenged birds suggest that this pathway is not activated in prebiotic-treated birds regardless of *Salmonella* challenge. The difference in basal expression of these genes suggests a different level of stimulation by the intestinal contents, and that the difference in *Salmonella* load in the cecum could be due to changes in the microbiome.

The addition of GOS to the diet led to a significant difference in microbiome compared with the control birds at all-time points investigated. Control birds remained distinct from the other groups suggesting that both *Salmonella* challenge and prebiotic or + infected-prebiotic modified the chicken cecum microbiome. The control challenge and prebiotic challenge groups were not significantly different starting from 7 dpi, suggesting that while the microbiome was initially different challenging the birds with *Salmonella* lead to a similar microbiome regardless of bird treatment background. Changes in cecum microbiome have been reported in birds given candidate *Salmonella* vaccine strains (37), and infection with *Salmonella* has also been shown to modify the natural development of the chicken microbiota (38). The control un-challenged birds remain distinct from the other treatment groups throughout.

Statistical analysis of the composition of the non-infected age-matched birds showed significant differences in the composition over all time points investigated. In our study, there was a decrease in the level of *Clostridiales Ruminococcaceae* significantly decreased in the prebiotic-treated birds at 4, 7, and 27 dpi; a decrease in the level of these bacteria was also reported by Videnska et al. (16) and Mon et al. (38) in the cecum of after *Salmonella* challenge. *Clostridiales Lachnospiraceae* was significantly increased at 4, 7, and 12 dpi and significantly reduced at 27 dpi in prebiotic-treated birds compared with control-treated birds. *Clostridiales* showed a significant decrease at 4 dpi and a significant increase at 12 dpi in prebiotic-treated birds compared with control-treated birds. *Lactobacillus* was significantly lower at 7 dpi and significantly increased at 12 and 27 dpi. *Lactobacilli* isolated from chickens have been shown to be inhibitory to the growth of *Salmonella* (39), and the increased presence of *Lactobacillius* in the chicken cecum seen from 12 dpi may play a role in the reduction in *Salmonella* CFU/g cecum content seen in the challenged birds. In this study, the development of the microbiome plays a role in the cecum immune response during a *Salmonella* challenge; the rate of the development of the microbiome is also influenced by the addition of GOS and *Salmonella* challenge. The changes in gene expression seen during treatment with GOS and/or *Salmonella* challenge along with the difference in clearance rates between the two *Salmonella* serovars may be driven by the changes in the microbiome. Further analysis of the changes in the microbiome will be the focus of future studies.

Taken together, these changes in gene expression indicate that there is an underlying effect on the birds with the addition of GOS and these changes result in a reduction in the level of immune response when birds are challenged with *Salmonella* at day 3 of life. While these changes result in a reduction in gene expression they correspond with changes in the microbiome, specifically an increase in the level of *Lactobacillales* and a decrease in *Clostridiales* suggest that these changes may affect *Salmonella*’s ability to colonize birds. The data presented here demonstrate that GOS can be used to cause subtle changes the gene expression in both the TLR4 and MYD88 pathways, and more substantive changes in the microbiome. This is consistent with other studies where the addition of GOS leads to an increase in *Bifidobacteria* and *B. lactis* in chickens (21), and suggests that GOS along with other prebiotics can be used to modify the intestinal ecosystem, and in turn the host immune response. The specific changes within the microbiome induced by GOS are beyond the scope of this current study. Our lab is currently investigating the specific microbiome and metabolomic changes induced by GOS to better decipher the mechanisms of the changes in gene expression, and microbiome changes seen in this current study.

## Ethics Statement

This study was carried out in accordance to the recommendations in the Guide for Care and Use of Laboratory Animals of the National Institutes of Health. The protocol was approved by the Institutional Animal Care and Use Committee (IACUC) (Protocol no. OLAW D16-00214) protocol (Protocol no. 15–065-A).

## Author Contributions

R-AH contributed to the sample collection, real-time PCR data collection, data analysis, and writing of the manuscript. RA contributed to the sample collection, data analysis, and writing of the manuscript. MM contributed to the sample collection, microbiology data collection, microbiology data analysis, microbiome data analysis, and writing of the manuscript. HH and MK contributed to the experimental design of the study and writing of the manuscript.

## Conflict of Interest Statement

The authors declare that the research was conducted in the absence of any commercial or financial relationships that could be construed as a potential conflict of interest. The reviewers CS and CL and handling editor declared their shared affiliation.

## Footnotes

^1^http://www.pathwaycommons.org/about.

^2^https://www.med.unc.edu/microbiome.
